# Draft Genome Sequence of the *Lactobacillus agilis* Strain Marseille

**DOI:** 10.1128/genomeA.00840-15

**Published:** 2015-07-30

**Authors:** Fatima Drissi, Noémie Labas, Vicky Merhej, Didier Raoult

**Affiliations:** Aix Marseille Université, CNRS 7278, Inserm 1095, URMITE, UM63, IRD 198, Marseille, France

## Abstract

We report the draft genome sequence of *Lactobacillus agilis* strain Marseille, isolated from stool samples of a child suffering from kwashiorkor. This strain can use two metabolic pathways allowing the assimilation of glucose and xylose. Here, we present the first draft genome of the *Lactobacillus agilis* species.

## GENOME ANNOUNCEMENT

*Lactobacillus agilis* strain Marseille is a lactic acid bacterium isolated from fecal samples of a child suffering from a severe form of malnutrition with insufficient protein consumption, called kwashiorkor. Glucose clearance rates are affected in children with kwashiorkor (1), and gut microbiome was found to be one of the causal factors in this disease (2). As it has the ability to ferment hexoses through the glycolysis pathway or use the pentose phosphate pathway for pentose assimilation (3), *Lactobacillus agilis* is a facultatively heterofermentative bacteria.

The genome of *Lactobacillus agilis* Marseille was sequenced with the mate pair strategy on the MiSeq technology (Illumina Inc., San Diego, CA, USA). The reads where assembled through Velvet software (4), and the contigs obtained were combined together by SSPACE (5), Opera software version 1.2 (6), and GapFiller version 1.10 (7). Noncoding genes and miscellaneous features were predicted using RNAmmer (8), ARAGORN (9), Rfam (10), Pfam (11), and Infernal (12), while coding DNA sequences (CDSs) were predicted using Prodigal (13). Functional annotation was achieved using the Rapid Annotation using Subsystem Technology (RAST) server and BLAST+ (14) against the KEGG and COG databases.

The draft genome is composed of 12 scaffolds and includes 2,369,669 bases (G+C content of 46.18%). It comprises 2,282 predicted genes, including 12 rRNAs (7 genes are 5S rRNA, 3 genes are 16S rRNA, and 2 genes are 23S rRNA) and 84 tRNAs genes. A total of 1,535 genes (69.80%) were assigned a putative function, 62 genes were identified as ORFans (2.82%), and the 481 remaining genes were annotated as hypothetical proteins (21.87%). The 16S rRNA analysis showed strong homology to other *Lactobacillus* species with completed genomes, including *Lactobacillus salivarius* JCM1046 (CP007646.1), with 95% similarity, and *Lactobacillus ruminis* ATCC 27782 (CP003032.1), with 94% similarity. The draft genome sequence of strain Marseille is larger than those of *L. ruminis* and *L. salivarius* (2.07 Mb and 2.31 Mb, respectively), and its G+C content is larger than those of *L. ruminis* and *L. salivarius* (43.50% and 32.97%, respectively).

The genome of strain Marseille encodes for two fructose-1,6-diphosphate aldolases (EC 4.1.2.13) involved in the glycolysis pathway and one phosphoketolase (EC 4.1.2.9) involved in the pentose phosphate pathway. According to the carbon source, strain Marseille can use both glycolysis and pentose phosphate pathways, and thus can produce either acetic acid, formic acid, and ethanol from sugars, or lactic acid under glucose limitation. Moreover, the genome of strain Marseille encodes for several enzymes that participate in carbohydrate transport and metabolism and two enzymes (acetyl-CoA acetyltransferase and carboxylesterase type B) involved in lipid transport and metabolism that are not present in *L. ruminis* ATCC 27782 and *L. salivarius* JCM1046. These specific features of *L. agilis* may result from a strategy of the bacterium to adapt to the particular environment of severely malnourished children and/or may have a causal effect in the development of the disease.

### Nucleotide sequence accession number.

This whole-genome shotgun project has been deposited in ENA under the accession number CVQY00000000.
